# Deciphering the Dysregulated Pathways and Candidate Therapeutic Compounds for Primary Ovarian Cancer Using Whole Transcriptomics Data and Next Generation Knowledge Discovery Strategies

**DOI:** 10.1111/jcmm.71356

**Published:** 2026-09-22

**Authors:** Peter Natesan Pushparaj, Kalamegam Gauthaman, Alaa G. Alahmadi, Reem Nabil Hassan, Hind A. Alkhatabi, Ammar AL‐Farga

**Affiliations:** ^1^ Institute of Genomic Medicine Sciences, Faculty of Applied Medical Sciences King Abdulaziz University Jeddah Saudi Arabia; ^2^ Center for Transdisciplinary Research, Department of Pharmacology, Saveetha Dental College and Hospitals Saveetha Institute of Medical and Technical Sciences Chennai India; ^3^ Department of Biological Science, College of Science University of Jeddah Jeddah Saudi Arabia; ^4^ Department of Biological Sciences, Faculty of Sciences‐ King Abdulaziz University Jeddah Saudi Arabia

**Keywords:** ExpressAnalyst, L1000CDS2, L1000FWD, ovarian cancer, precision medicine, RNAseq, WebGestalt

## Abstract

Ovarian cancer (OC) is a type of gynaecological cancer with a higher mortality rate due to diagnosis at an advanced stage and limited treatment options. This study aimed to leverage transcriptomic data to identify cellular and molecular pathways and potential anti‐cancer compounds that specifically target primary invasive epithelial ovarian cancer (EOC). By employing next‐generation knowledge discovery (NGKD) methodologies, we sought to unravel the intricate molecular landscape of primary invasive EOC using RNA sequencing (RNA‐seq) data and decipher potential therapeutics for this debilitating disease. We performed NGKD analysis of the Gene Expression Omnibus (GEO) dataset GSE1295399 obtained from whole RNA‐seq experiments. Using the raw counts and filtered metadata from GEO, we identified 2123 differentially expressed genes (DEGs) based on a Log_2_ fold change (≤ ±0.6) and a *p*‐value cutoff of < 0.05, between primary invasive EOC and benign EOC using the ExpressAnalyst platform. The DEGs were further analysed using both ExpressAnalyst and WebGestalt tools for differentially regulated cellular and molecular pathways and gene ontology (GOs), including biological process (GO‐BP), molecular function (GO‐MF) and cellular components (GO‐CC). Both L1000 Fire Works Display (L1000FWD) and L1000 Characteristic Direction Signature Search Engine (L1000CDS2) tools were used to decipher synthetic or natural chemical compounds with the potential to reverse OC‐associated gene signatures. DEGs implicated in key cellular and molecular pathways, such as oxidative phosphorylation, cell cycle, proteasome, programmed cell death protein‐1 (PD‐1) signalling, nuclear factor kappa B (NF‐kB) signalling, cytokine and chemokine signalling, natural killer cell‐mediated cytotoxicity and microRNAs in cancer, were positively enriched. The ribosome, translation, translational initiation and elongation and transforming growth factor‐beta (TGF‐β) signalling were negatively enriched in primary invasive EOC. Based on NGKD analysis, we identified approximately 50 synthetic or natural compounds, including naproxol, palbociclib, etoposide, wortmannin, PP‐110, AZD‐8055, amsacrine and BRD‐K6595526. The results of this study could aid in the development of personalized treatment plans based on the unique profile of each tumour type, thus facilitating the development of personalized or precision treatment plans and improving diagnostic and prognostic capabilities in the clinic. In conclusion, the combination of RNA‐seq and cutting‐edge NGKD methodologies holds significant promise for identifying key cellular and molecular pathways and OC therapeutics.

## 1 Introduction

**FIGURE 1:**
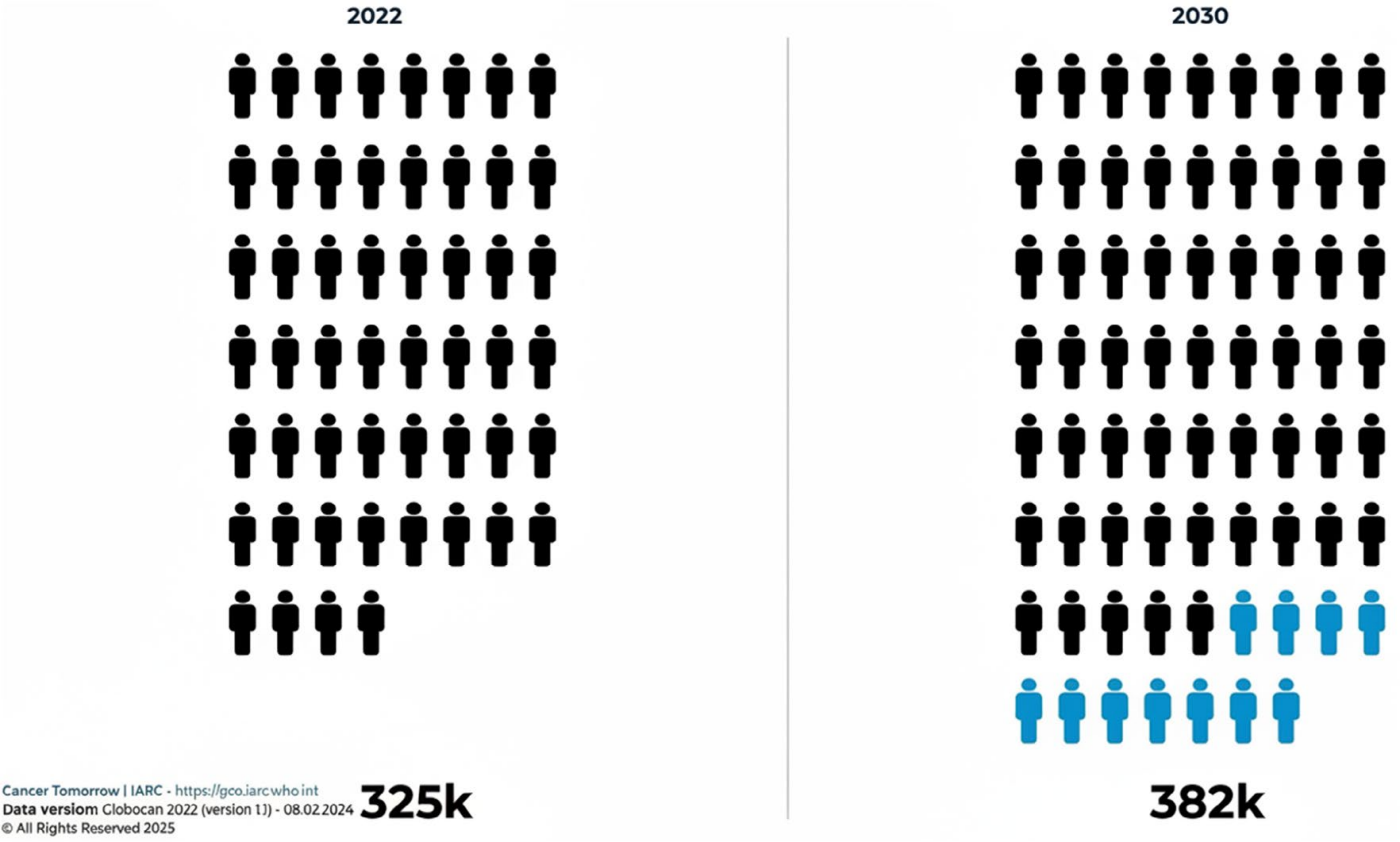
GLOBOCAN 2022 statistics. By 2030, the global incidence of OC among women (age 0–85 years) will exhibit a notable escalation of approximately 17.54% from 2022, resulting in a total of 381,561 new diagnoses (Globocan 2022 [version 1.1]—08.02.2024, https://gco.iarc.who.int, accessed on 3 September 2025).

Ovarian cancer (OC) is the second most common gynaecological malignancy, with the highest mortality rate [1]. According to the GLOBOCAN 2020 database of the International Agency for Research on Cancer (IARC), OC is the eighth most common cancer in women and the seventh most common cause of cancer‐related death in women [1]. In 2020, it was estimated a worldwide age‐standardized incidence rate (ASR) of OC of 6.6 per 100,000 and a worldwide age‐standardized mortality rate of 4.2 per 100,000 individuals (Globocan 2022 [version 1.1]—08.02.2024, https://gco.iarc.who.int, accessed on 3 September 2025). The risk of developing OC increases with age and ranked 7th among Saudi women based on 2008 statistics [2]. In KSA, 444 new cases and 281 deaths were reported in 2020, accounting for 1.6% of all cancer cases, with an ASR of 3.8 per 100,000 and a mortality rate of 2.7 per 100,000 [3]. It is anticipated that by the year 2030, the global incidence of OC among women will exhibit a notable increase of approximately 17.54% from 2022, resulting in a total of 381,561 new diagnoses. Moreover, the annual toll of fatalities attributed to OC is expected to increase significantly, surging by over 50% compared to the statistics of 2020, reaching a projected count of 313,617 deaths (https://gco.iarc.who.int/tomorrow/en/dataviz/isotype) (Figure 1).

**FIGURE 2:**
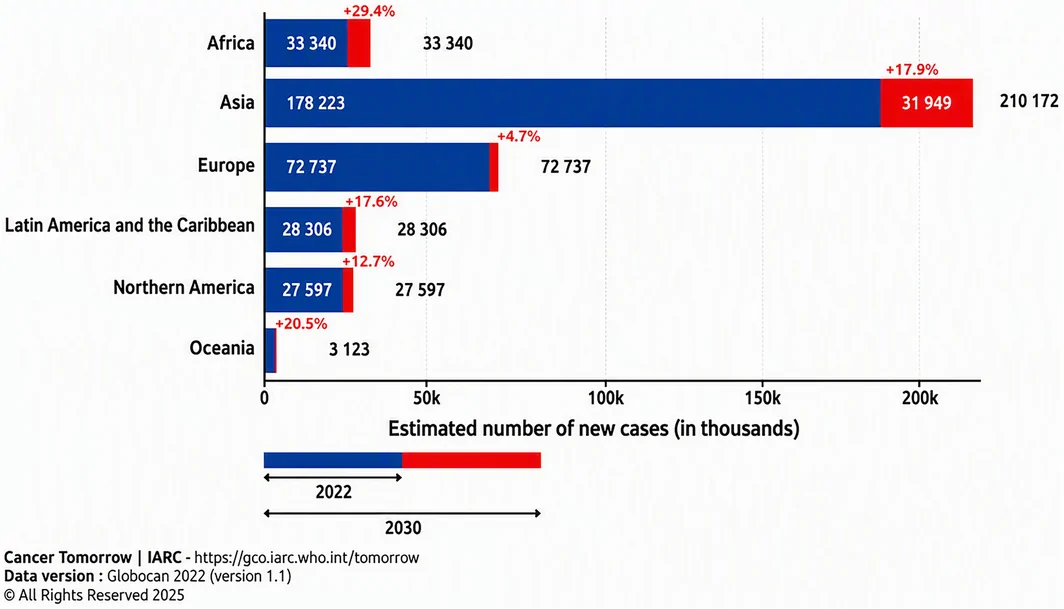
GLOBOCAN 2022 statistics. Estimated number of new cases from 2022 to 2030 (age 0–85 years) on different continents (Globocan 2022 [version 1.1]—08.02.2024, https://gco.iarc.who.int, accessed on 3 September 2025).

Globally, approximately 207,000 women die from OC annually [4, 5], and the estimated number of cases in different continents is predicted to increase by 2030 (https://gco.iarc.who.int/tomorrow/en/dataviz/bars) (Figure 2). Epithelial OC, which represents approximately 90%–95% of all OC cases, is frequently diagnosed at an advanced stage, with over 70% of patients presenting with advanced disease at the time of initial diagnosis and a 5‐year survival rate below 30%. It is a complicated and multifaceted disease, making early detection difficult, as it is often asymptomatic until later stages, [5, 6].

Genetic and environmental factors play important roles in OC development. In addition, epigenetic changes and mutations contribute significantly to the pathogenesis of OC and influence diagnosis and treatment strategies [1]. The symptoms of OC include abdominal, pelvic and stomach bloating; pain; rapid distension; and problems urinating, which make the diagnosis of OC more difficult [7]. Currently, OC treatment usually consists of a combination of cytoreductive surgery and platinum‐based chemotherapy. However, novel therapeutic approaches are needed, as the incidence and mortality of OC are predicted to increase worldwide [8]. Targeting metabolic pathways and the tumour microenvironment (TME) may also improve OC treatment outcomes [9].

Transcriptomic profiling has often been used as a key high‐throughput omics technique to decipher the precise cellular and molecular mechanisms involved in OC [10]. Bulk RNA sequencing (bulk RNA‐seq) or whole RNA sequencing is the most widely used approach for large‐cohort clinical studies and biobank projects because of its robustness, high transcriptome coverage and cost‐effectiveness. It offers comprehensive coverage of gene expression across heterogeneous tissue samples and is useful for capturing important cellular and molecular signatures and major dysregulated pathways that cause the overall phenotype of a sample [11]. RNAseq is especially useful for finding drugs for repurposing based on the gene signatures of a disease that therapeutic agents target to generate a broad, clinically relevant response. Thus, utilization of existing high‐quality whole transcriptomic datasets provides a powerful foundation for identifying disease‐specific gene signatures and dysregulated pathways suitable for deciphering candidate therapeutic compounds [12].

Hence, in this study, we used next‐generation knowledge discovery (NGKD) tools to examine whole transcriptome data from primary invasive EOC to identify differentially regulated cellular and molecular signalling pathways and decipher candidate anticancer compounds. These results could aid in the development of personalized treatment plans based on unique OC gene expression profiles. In addition, the NGKD methods used in this study could be relevant to other cancers and could expand the field of personalized medicine and targeted cancer therapies.

## 2 Materials and Methods

### 2.1 Ethics Statement

This study did not require Institutional Review Board (IRB) approval because we did not use animal models or human subjects, and the data was already de‐identified. This study relied on RNA‐seq datasets obtained from Gene Expression Omnibus (GEO) [13]. Ethical approval was obtained during sample collection, further processing, and submission of data to Gene Expression Omnibus (GEO) [14]; thus, the data can be freely accessed and reanalyzed using NGKD tools [15].

### 2.2 Data Source

High‐throughput whole RNA‐seq data (RNA‐seq) were obtained from the GEO database (accession number GSE295399). These data were collected from patients with benign (*n* = 4), borderline (*n* = 17), and malignant epithelial ovarian tumours (Stages III and IV) (*n* = 125) [14]. As previously described, ExpressAnalyst was used to clarify the data by removing outliers and analysing the raw gene‐level counts and metadata for this dataset [15].

### 2.3 High‐Dimensional Data Analysis Using ExpressAnalyst

ExpressAnalyst is an open‐source tool for the analysis of RNA‐seq data (https://www.expressanalyst.ca/) (accessed on 19 June 2026) [16, 17]. Using the raw counts and filtered metadata, we identified 2123 differentially expressed genes (DEGs) based on Log_2_ fold change (≤ ±0.6) and a *p*‐value cutoff of < 0.05, between primary invasive EOC cells and benign EOC (File S1), and analysed as previously described [15].

### 2.4 WebGestalt Analysis

DEGs derived from primary invasive EOC compared to benign EOC were analysed using the WebGestalt tool (wGSEA) [15, 18, 19] (accessed on 21 June 2026), an open‐source software platform (https://www.webgestalt.org/) specifically designed for Gene Set Enrichment Analysis (GSEA) of several mammalian species, including *Homo sapiens* . Pathway analysis was performed using Wikipathways and Gene Ontology (GO) enrichment analysis, providing insights into the biological processes (GO‐BP), molecular functions (GO‐MF), and cellular components (GO‐CC) affected in primary invasive EOC. As described previously, the reference list for each analysis included all mapped gene symbols from the selected platform genome, with the parameters for the enrichment analysis set at a minimum of three and a maximum of 2000 IDs in the category, and a false discovery rate (FDR) of *p* < 0.05, computed using the Benjamini–Hochberg (BH) method [15].

### 2.5 L1000FWD And L1000CDS2 Analyses

DEGs derived from primary invasive EOC compared to benign EOC were analysed using the L1000 Fire Works Display (L1000FWD) to identify the top 50 drugs and natural products with the potential to reverse OC‐associated gene signatures [20, 21]. The L1000 Characteristic Direction Signature Search Engine (L1000CDS2) was used to identify the top 50 drugs and natural products with the potential to reverse OC‐associated gene signatures [22]. These NGKD tools help decode large‐scale gene expression data to identify novel or known compounds with therapeutic potential for treating primary OC, as previously described [15].

## 3 Results

**FIGURE 3:**
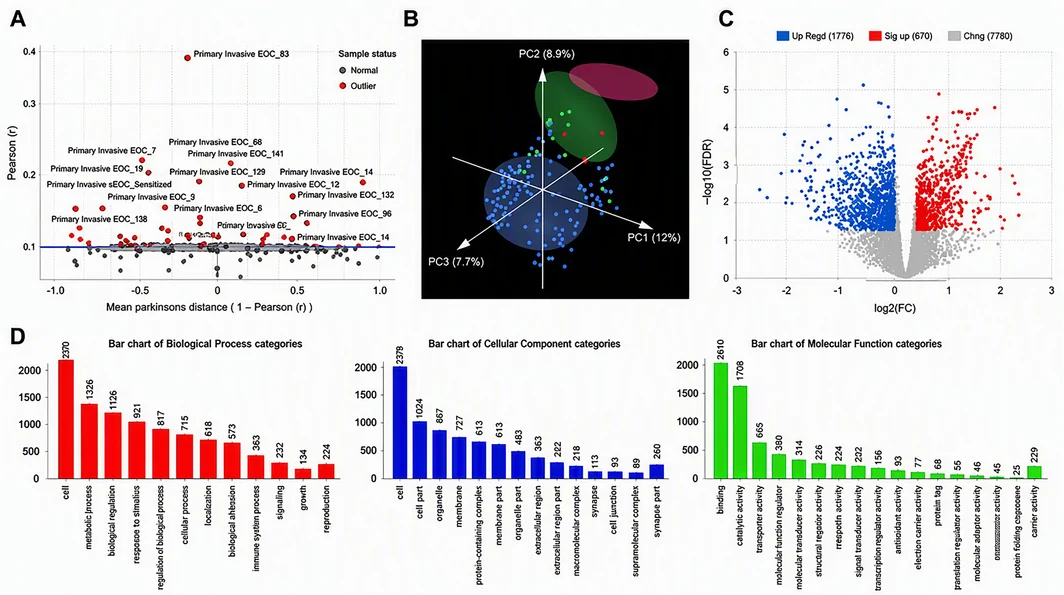
ExpressAnalyst analysis. (A) Identification of outliers based on dendrogram threshold. (B) Principal Component Analysis (PCA) of benign, moderate and primary invasive EOC samples. (C) Volcano plot showing the DEGs in primary invasive EOC compared to benign EOC. (D) GO Slim bar charts showing the Biological, Cellular Component and Molecular Function categories based on the DEGs derived by comparing primary EOC with benign EOC.

Here, we performed NGKD analysis of the GEO dataset GSE295399 obtained from RNA‐seq experiments to identify DEGs in primary invasive EOC compared to benign EOC. The outliers were filtered based on a dendrogram threshold of > 0.1 (Figure 3A), and principal component analysis (PCA) was performed using ExpressAnalyst (Figure 3B). Using the raw counts and filtered metadata, we identified 2123 DEGs based on a *p*‐value cutoff of < 0.05 and Log2 Fold Change (≤ ±0.6) between primary invasive EOC and benign EOC, as depicted in the volcano plot (Figure 3C). The number of DEGs involved in various biological processes, cellular components and molecular functions was determined using GO Slim analysis based on WebGestalt (Figure 3D).

**FIGURE 4:**
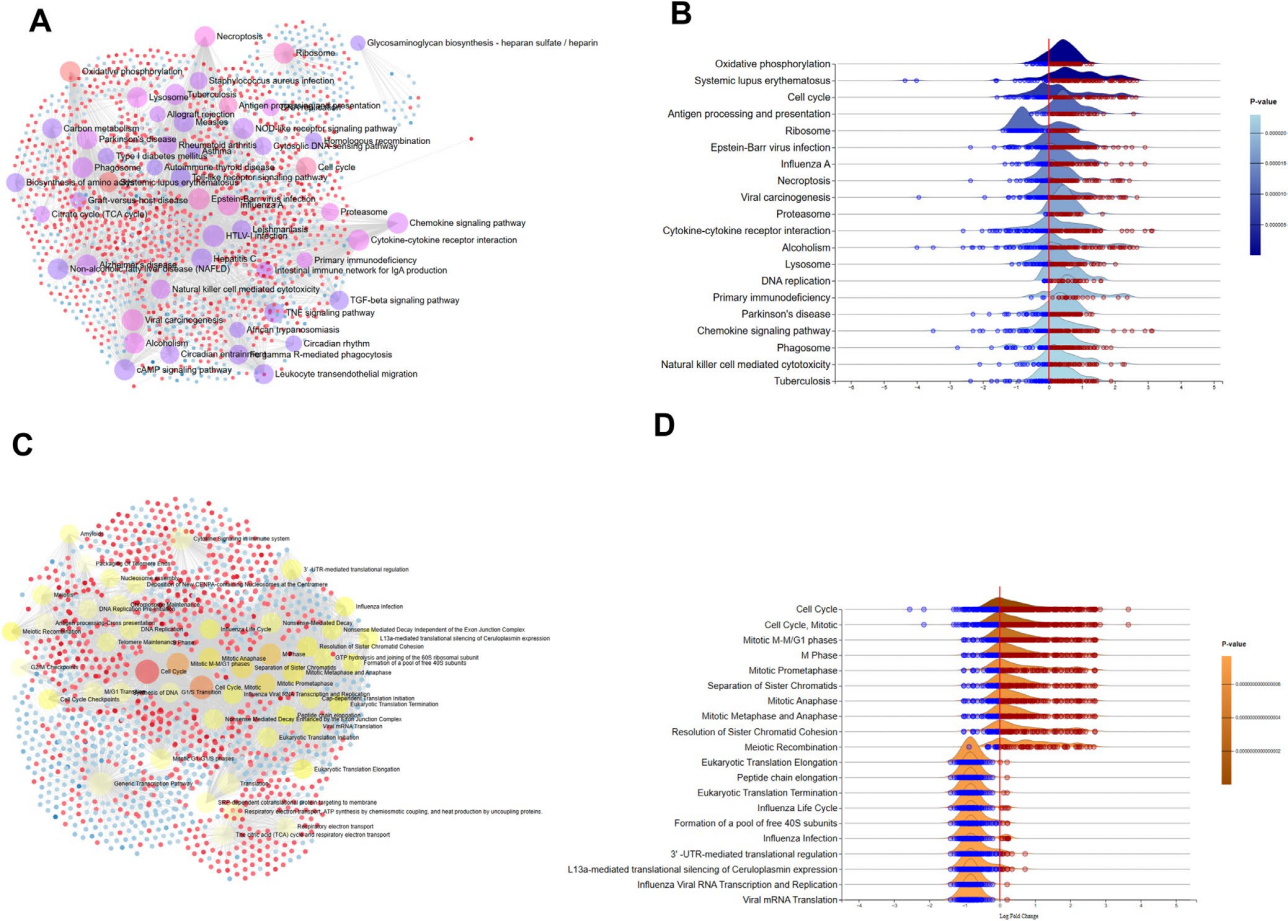
Differentially regulated cellular and molecular pathways based on ExpressAnalyst analysis. (A) Bipartite network of differentially regulated KEGG pathways. (B) Ridgeline plot of differentially regulated KEGG pathways. (C) Bipartite network of differentially regulated Reactome pathways. (D) Ridgeline plot of differentially regulated Reactome pathways obtained using DEGs obtained from primary invasive EOC compared to benign EOC.

Bipartite network and ridgeline plot analyses based on KEGG pathways showed that oxidative phosphorylation, cell cycle, proteasome, DNA replication and chemokine signalling were positively enriched in primary invasive EOC, whereas DEGs implicated in ribosome signalling were negatively enriched (Figure 4A,B). Similarly, bipartite network and ridgeline plot analyses based on Reactome pathways showed that the cell cycle, mitosis, and separation of sister chromatids were positively enriched, whereas eukaryotic translation initiation, translation elongation, translation termination, and 3′‐UTR‐mediated translational regulation were negatively enriched in primary invasive EOC (Figure 4C,D).

**FIGURE 5:**
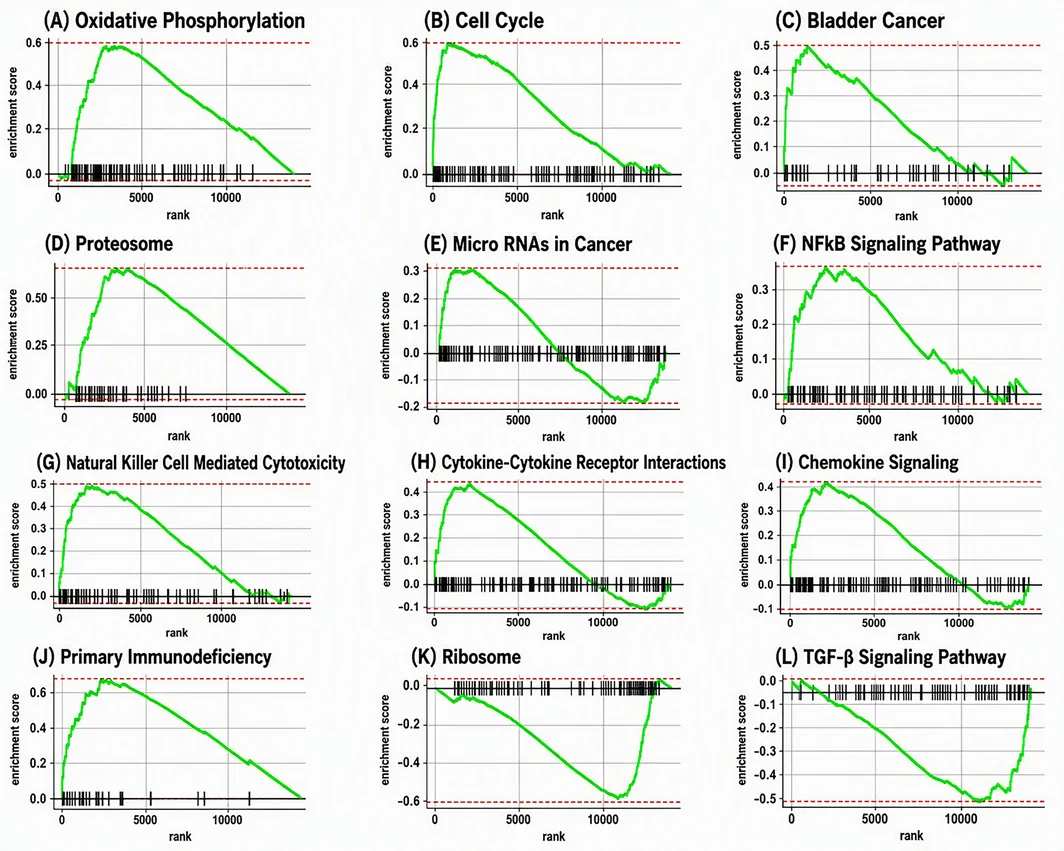
Gene Set Enrichment Analysis (GSEA) was performed based on KEGG pathways using ExpressAnalyst. Positive enrichment of (A) oxidative phosphorylation, (B) cell cycle, (C) bladder cancer, (D) proteasome, (E) microRNAs in cancer, (F) NF‐κB signalling, (G) natural killer cell‐mediated cytotoxicity, (H) cytokine‐cytokine receptor Interactions, (I) chemokine signalling, (J) primary immunodeficiency, and negative enrichment of (K) ribosome and (L) TGF‐β signalling pathways based on DEGs obtained from primary invasive EOC compared to benign EOC.

**FIGURE 6:**
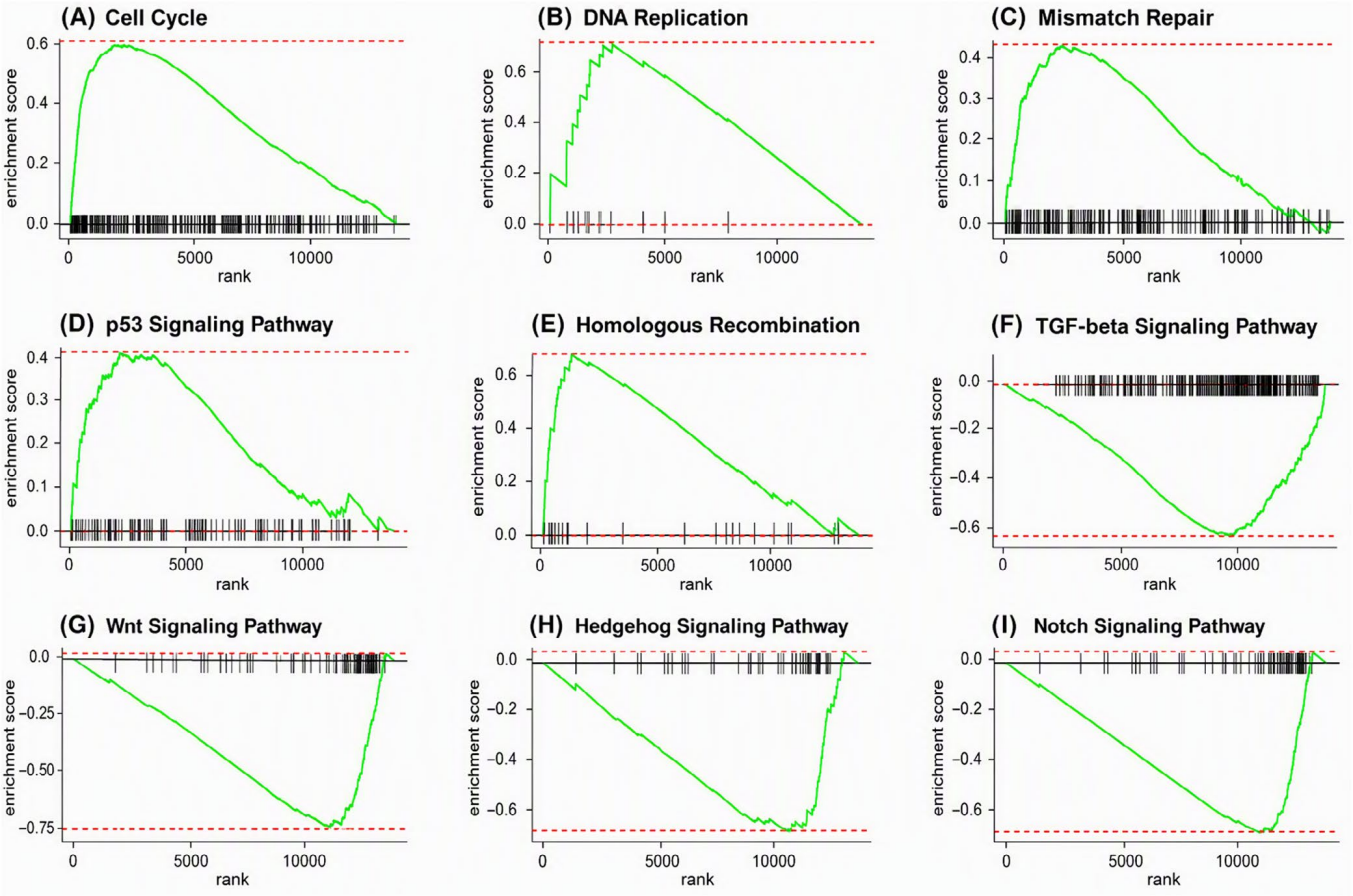
Gene Set Enrichment Analysis (GSEA) was performed based on Reactome pathways using ExpressAnalyst. Positive enrichment of (A) cell cycle (B) PD‐1 signalling (C) cytokine signalling in immune system (D) signalling by Interleukins (E) chemokine receptors bund chemokines and the negative enrichment of (F) generic transcription pathway (G) eukaryotic translational initiation (H) translation initiation complex formation and (I) 3′‐UTR mediated translational regulation based on DEGs obtained from primary invasive EOCcompared to benign EOC.

Furthermore, the GSEA analysis of DEGs based on KEGG showed that oxidative Phosphorylation, cell cycle, bladder cancer, proteosome, microRNAs in cancer, NFkB signalling, natural killer cells mediated cytotoxicity, cytokine‐cytokine receptor interactions, chemokine signalling, primary immunodeficiency and the negative enrichment of ribosome and TGF‐β signalling pathways (Figure 5). GSEA of DEGs based on Reactome showed that the cell cycle, PD‐1 signalling, cytokine signalling in the immune system, signalling by interleukins and chemokine receptor binding chemokines were positively enriched and the generic transcription pathway, eukaryotic translational initiation, translation initiation complex formation, and 3′‐UTR‐mediated translational regulation were negatively enriched based on DEGs obtained from primary invasive EOC compared to benign EOC (Figure 6).

**FIGURE 7:**
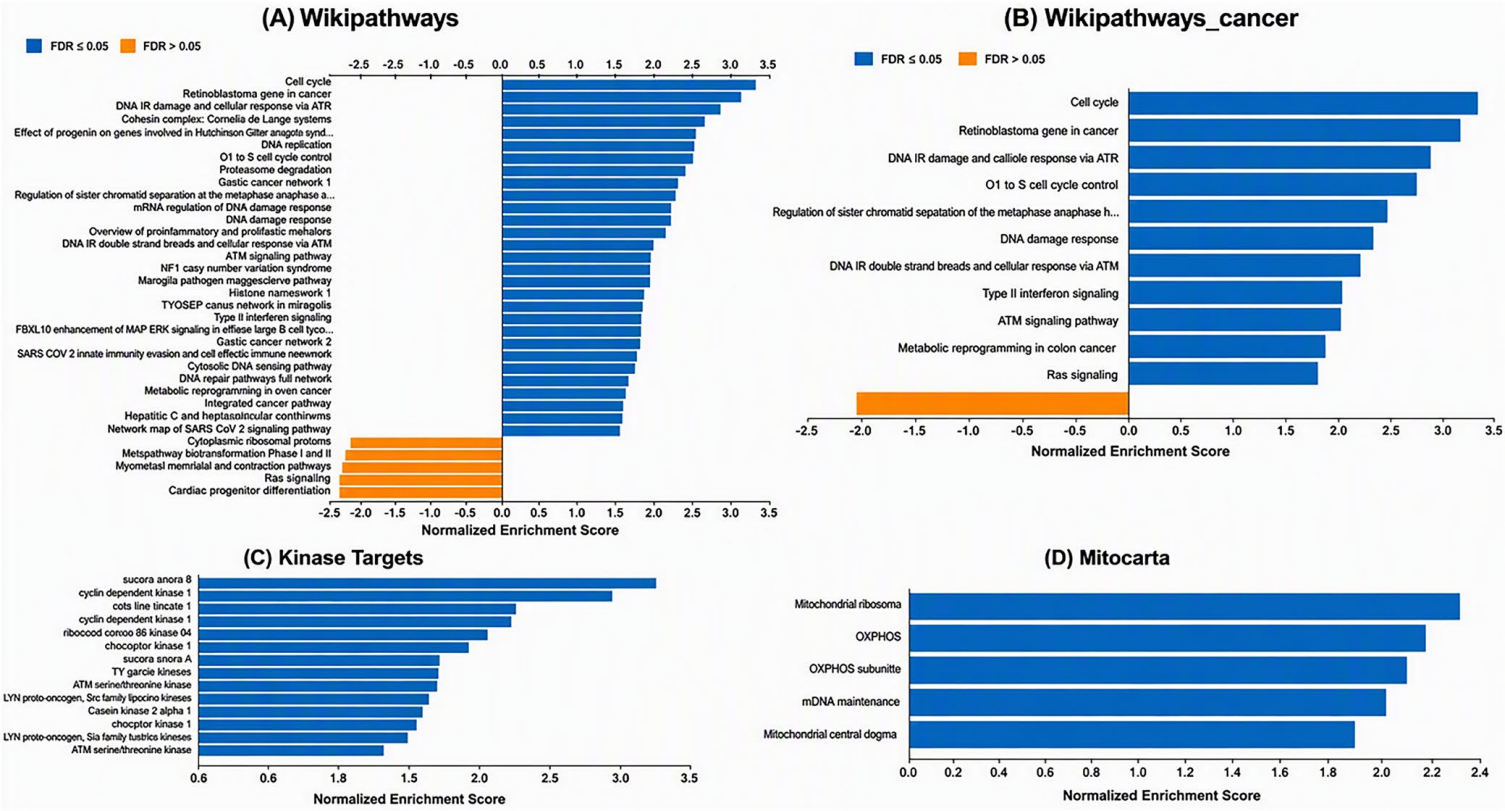
WebGestalt analysis. (A) Enrichment of cellular and molecular pathways based on (A) WikiPathways, (B) WikiPathways_cancer, (C) kinases and (D) Mitocarta using DEGs obtained from primary invasive epithelial ovarian cancer (EOC) compared to benign EOC.

WebGestalt analysis of DEGs derived from primary invasive EOC based on WikiPathways revealed that the Cell cycle, Proteasome degradation, G1 to S cell cycle control, DNA replication, Regulation of sister chromatid separation at the metaphase anaphase transition, miRNA regulation of DNA damage response, Overview of proinflammatory and profibrotic mediators, etc. were positively enriched, whereas the Cytoplasmic ribosomal proteins were negatively enriched in primary invasive EOC (Figure 7A). WebGestalt analysis of DEGs derived from primary invasive EOC based on WikiPathways_Cancer revealed that Cell cycle, G1 to S cell cycle control, Regulation of sister chromatid separation at the metaphase anaphase transition, DNA damage response, ATM signalling pathway, Type II interferon signalling were positively enriched, whereas Ras signalling was negatively enriched (Figure 7B). WebGestalt analysis further showed that the genes implicated in kinase networks such as ATM serine/threonine kinase, ATR serine/threonine kinase, aurora kinase A, aurora kinase B, cyclin‐dependent kinase 1, cyclin‐dependent kinase 2, checkpoint kinase 1, checkpoint kinase 2, casein kinase 2 alpha 1, LCK proto‐oncogene, Src family tyrosine kinase, LYN proto‐oncogene, Src family tyrosine kinase, polo‐like kinase 1, ribosomal protein S6 kinase A4, and TTK protein kinase were positively enriched in primary invasive EOC (Figure 7C). Similarly, we observed based on Mitocarta, genes implicated in Mitochondrial ribosome OXPHOS, mtDNA maintenance, OXPHOS subunits and Mitochondrial central dogma were positively enriched in primary invasive EOC (Figure 7D). Additional results using NGKD analysis of DEGs for gene ontologies based on GO (Figure S1) and Panther (Figure S2) using ExpressAnalyst and KEGG, Reactome, Panther and GO databases using WebGestalt (Figure S3) are provided in File S1.

**FIGURE 8:**
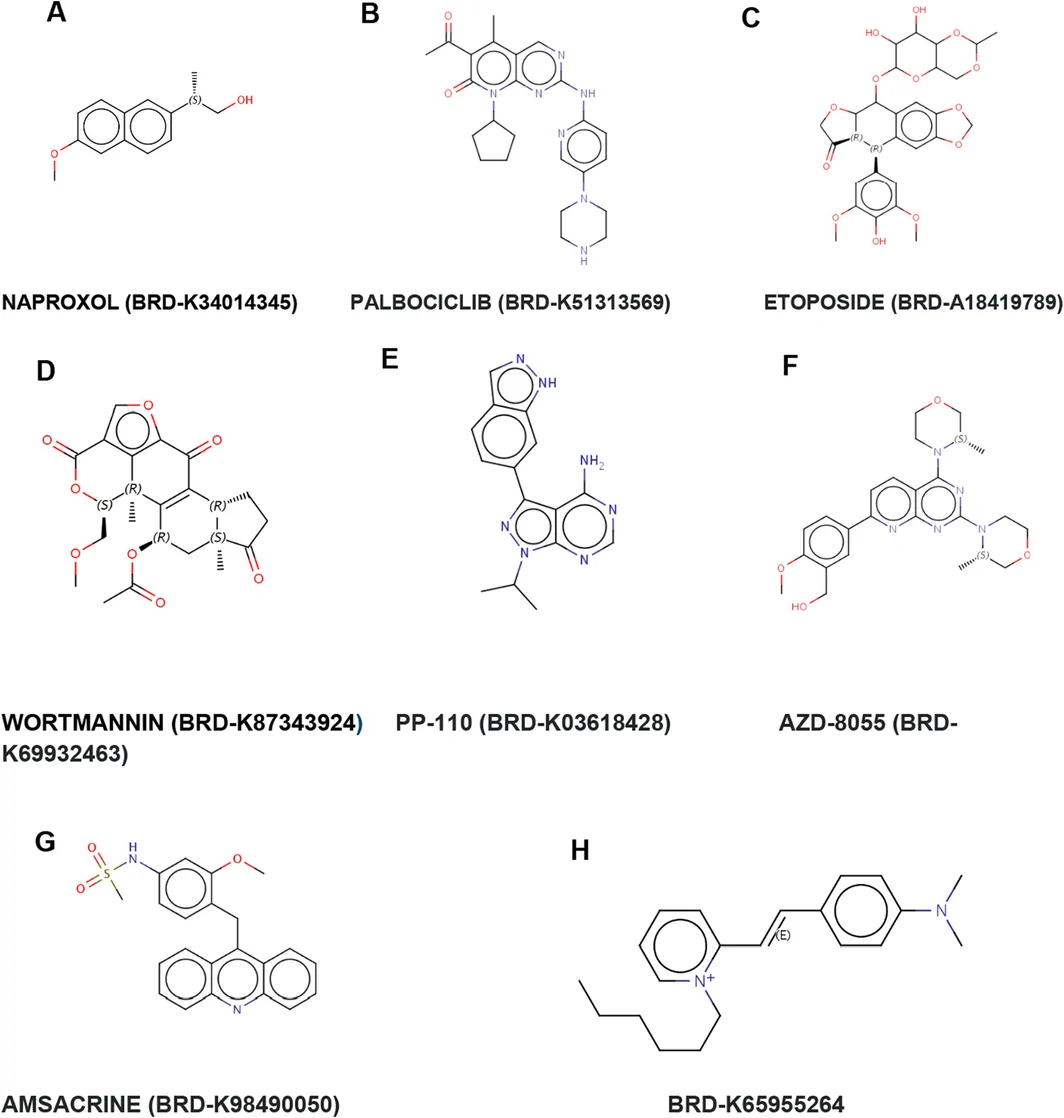
L1000FWD and L1000CDS2 analyses of DEGs derived from primary invasive EOC compared to benign EOC revealed many compounds with the potential to ameliorate primary invasive EOC. Some of these compounds are (A) NAPROXOL (BRD‐K34014345), (B) PALBOCICLIB (BRD‐K51313569), (C) ETOPOSIDE (BRD‐A18419789), (D) WORTMANNIN (BRD‐K87343924), (E) PP‐110 (BRD‐K03618428), (F) AZD‐8055 (BRD‐K69932463), (G) AMSACRINE (BRD‐K98490050) and (H) BRD‐K65955264.

We identified the top 50 drugs or natural products with the potential to reverse DEGs associated with primary invasive EOC. We identified that naproxol (brd‐k34014345), palbociclib (BRD‐K51313569), etoposide (BRD‐A18419789), wortmannin (BRD‐K87343924), PP‐110 (BRD‐K03618428), AZD‐8055 (BRD‐K69932463), and BRD‐K65955264 (Figure 8) had significant effects (*p* < 0.01; *Z*‐score < 1.7; combined score > −25) on the gene signatures derived from primary invasive the EOC using L1000FWD tool (Table 1). Furthermore, we used the L1000CDS2 tool to identify the top 50 drugs or natural products, including palbociclib (BRD‐K51313569), wortmannin (BRD‐K87343924), PP‐110 (BRD‐K03618428), AZD‐8055 (BRD‐K69932463), amsacrine (BRD‐K98490050) and BRD‐K65955264 (Figure 8) which could reverse the expression of DEGs associated with primary invasive EOC (Table 2).

**TABLE 1:** Top 50 reverse gene signatures based on L1000FWD tool obtained using the DEGs in primary invasive EOC compared to benign EOC.

sig_id	Drug	Similarity score	*p*	*q*	*Z*‐score	Combined score
CPC015_MCF7_24H:BRD‐K34014345‐001‐03‐4:10	naproxol	−0.0973	1.06E‐45	4.54E‐41	1.69	−75.98
CPC006_VCAP_24H:BRD‐K69932463‐001‐03‐1:10	AZD‐8055	−0.0889	5.73E‐39	4.09E‐35	1.77	−67.69
CPC010_MCF7_24H:BRD‐K65955264‐005‐03‐8:10	BRD‐K65955264	−0.0874	1.07E‐37	6.57E‐34	1.74	−64.18
LJP001_BT20_24H:BRD‐K51313569‐001‐03‐7:2	palbociclib	−0.0927	4.60E‐40	3.94E‐36	1.61	−63.14
LJP001_MDAMB231_24H:BRD‐K51313569‐001‐03‐7:10	palbociclib	−0.0828	4.16E‐37	1.98E‐33	1.68	−61.14
CPC005_MCF7_24H:BRD‐A18419789‐001‐01‐4:10	etoposide	−0.0812	4.62E‐34	1.41E‐30	1.83	−60.97
CPC015_MCF7_24H:BRD‐K98490050‐001‐01‐8:10	amsacrine	−0.0874	1.12E‐36	4.78E‐33	1.69	−60.62
CPC006_HCC515_24H:BRD‐A52650764‐001‐01‐0:10	ingenol	−0.0843	6.05E‐33	1.52E‐29	1.75	−56.49
LJP001_MDAMB231_24H:BRD‐K51313569‐001‐03‐7:2	palbociclib	−0.0797	8.04E‐34	2.29E‐30	1.66	−54.88
CPC015_MCF7_24H:BRD‐A34751532‐001‐04‐4:10	homosalate	−0.0812	3.16E‐31	6.75E‐28	1.68	−51.32
CPC004_VCAP_24H:BRD‐K87343924‐001‐02‐4:10	wortmannin	−0.0743	2.56E‐28	3.91E‐25	1.83	−50.47
LJP001_BT20_24H:BRD‐K51313569‐001‐03‐7:10	palbociclib	−0.082	6.99E‐32	1.59E‐28	1.61	−50.27
CVD001_HEPG2_24H:BRD‐K67439147‐001‐01‐7:2.5	SIB‐1893	−0.0782	4.28E‐30	8.32E‐27	1.67	−49.02
CPC017_MCF7_24H:BRD‐K05151076‐001‐01‐8:10	ZK‐164015	−0.0789	9.47E‐29	1.62E‐25	1.67	−46.8
CPC014_VCAP_24H:BRD‐U04166717‐000‐01‐2:10	XMD‐1499	−0.0728	3.65E‐27	4.88E‐24	1.72	−45.57
CPC008_A375_24H:BRD‐K30836161‐019‐01‐1:10	BRD‐K30836161	−0.0728	4.40E‐26	4.96E‐23	1.76	−44.62
CPC006_MCF7_24H:BRD‐A35588707‐001‐03‐0:1.25	teniposide	−0.072	5.93E‐25	5.90E‐22	1.75	−42.47
CPC006_VCAP_24H:BRD‐K21672174‐001‐02‐2:160	RO‐28‐1675	−0.0659	5.43E‐24	4.49E‐21	1.82	−42.3
CPC013_VCAP_24H:BRD‐K63068307‐001‐01‐4:10	ZSTK‐474	−0.0713	2.56E‐25	2.74E‐22	1.71	−42.03
CPC008_A375_24H:BRD‐K29506255‐019‐01‐0:10	BRD‐K29506255	−0.0697	2.50E‐24	2.38E‐21	1.78	−41.95
CPC018_MCF7_24H:BRD‐K67439147‐001‐01‐7:10	SIB‐1893	−0.072	1.22E‐25	1.34E‐22	1.67	−41.73
CPC006_VCAP_24H:BRD‐K67566344‐001‐01‐8:10	KU‐0063794	−0.0697	5.45E‐24	4.49E‐21	1.79	−41.63
CPC001_VCAP_24H:BRD‐K97365803‐001‐01‐3:10	PI‐828	−0.0697	1.94E‐23	1.46E‐20	1.82	−41.4
CPC009_MCF7_24H:BRD‐K00313977‐019‐01‐1:10	BRD‐K00313977	−0.0697	3.25E‐24	2.96E‐21	1.75	−41.18
CPC010_A549_24H:BRD‐K78385490‐019‐02‐2:10	BRD‐K78385490	−0.0697	2.85E‐24	2.65E‐21	1.74	−40.92
CPC014_MCF7_24H:BRD‐K51313569‐001‐02‐9:10	palbociclib	−0.0682	3.84E‐24	3.36E‐21	1.71	−40.1
CPC017_MCF7_24H:BRD‐K65814004‐003‐01‐1:10	diphenyleneiodonium	−0.0736	3.33E‐25	3.48E‐22	1.64	−40.02
CPC004_VCAP_24H:BRD‐A11678676‐001‐03‐4:10	wortmannin	−0.0651	4.49E‐22	2.87E‐19	1.83	−39.17
CPC014_VCAP_24H:BRD‐K49865102‐001‐02‐7:10	PD‐0325901	−0.0697	1.33E‐23	1.04E‐20	1.69	−38.72
CPC006_MCF7_24H:BRD‐A71390734‐001‐01‐7:0.08	idarubicin	−0.0682	4.64E‐22	2.92E‐19	1.77	−37.66
CPC016_MCF7_24H:BRD‐A35588707‐001‐03‐0:10	teniposide	−0.069	4.04E‐23	2.98E‐20	1.68	−37.55
LJP001_BT20_6H:BRD‐K51313569‐001‐03‐7:2	palbociclib	−0.0682	7.43E‐24	6.00E‐21	1.62	−37.46
CPC006_MCF7_24H:BRD‐K68548958‐001‐01‐2:20	BRD‐K68548958	−0.0636	2.49E‐21	1.48E‐18	1.82	−37.44
CPC006_HT29_24H:BRD‐K03618428‐001‐01‐3:22.2	PP‐110	−0.0697	5.10E‐22	3.16E‐19	1.73	−36.93
CPC017_MCF7_24H:BRD‐K14821540‐001‐03‐9:10	FCCP	−0.0682	2.54E‐22	1.70E‐19	1.65	−35.67
CPC001_HCC515_24H:BRD‐K59469039‐001‐03‐1:10	AG‐879	−0.0682	1.84E‐20	9.28E‐18	1.77	−34.85
CPD001_MCF7_24H:BRD‐K59456551‐001‐12‐7:10	methotrexate	−0.0659	1.08E‐21	6.63E‐19	1.66	−34.84
CPC006_HCC515_24H:BRD‐A15079084‐001‐02‐9:10	phorbol‐12‐myristate‐13‐acetate	−0.0674	2.15E‐20	1.07E‐17	1.75	−34.32
CPC016_MCF7_24H:BRD‐K94841585‐001‐02‐8:10	emodic‐acid	−0.0659	5.79E‐21	3.27E‐18	1.69	−34.21
CPC010_MCF7_24H:BRD‐A61856038‐001‐05‐3:10	tremulacin	−0.0644	2.17E‐20	1.07E‐17	1.74	−34.13
CPC006_HCC515_24H:BRD‐K68548958‐001‐01‐2:20	BRD‐K68548958	−0.0636	1.25E‐19	5.40E‐17	1.78	−33.6
CPC014_MCF7_24H:BRD‐K73293050‐001‐01‐5:10	WZ‐3146	−0.0659	3.25E‐20	1.57E‐17	1.7	−33.1
CPC019_A549_24H:BRD‐K31706415‐001‐01‐7:10	BRD‐K31706415	−0.0644	1.53E‐20	7.78E‐18	1.67	−33.08
CPC019_A549_24H:BRD‐K08448573‐001‐01‐3:10	BRD‐K08448573	−0.0659	8.22E‐21	4.40E‐18	1.64	−32.85
CPC017_MCF7_24H:BRD‐A62336480‐001‐02‐5:10	niguldipine	−0.0674	3.00E‐20	1.46E‐17	1.64	−31.99
CPC016_MCF7_24H:BRD‐K97365803‐001‐01‐3:10	PI‐828	−0.0636	2.74E‐19	1.16E‐16	1.68	−31.1
HOG003_A549_24H:BRD‐A71390734‐001‐01‐7:0.0412	idarubicin	−0.0659	8.88E‐20	4.04E‐17	1.59	−30.23
CPC017_MCF7_24H:BRD‐K59469039‐001‐03‐1:10	AG‐879	−0.0636	1.13E‐18	4.40E‐16	1.66	−29.85
ERG005_VCAP_48H:BRD‐A61304759‐001‐01‐0:5	tanespimycin	−0.0636	2.14E‐18	7.84E‐16	1.63	−28.76
CPC017_A549_24H:BRD‐A38030642‐001‐01‐2:10	cyclosporin‐a	−0.0636	3.62E‐18	1.28E‐15	1.64	−28.59

**TABLE 2:** Top 50 reverse gene signatures based on the L1000CDS2 tool obtained using the DEGs in primary invasive EOC compared to benign EOC.

Rank	Score	Perturbation	Cell‐line	Dose (μm)	Time (h)
1	0.1027	Ingenol 3, 20‐dibenzoate	HCC515	10.0	24
2	0.1027	NVP‐BEZ235	A549	0.37	24
3	0.1011	PP‐110	HT29	22.2	24
4	0.1011	GDC‐0980	A549	1.11	24
5	0.0981	PP‐110	A375	22.2	24
6	0.0981	Torin‐2	A549	0.12	24
7	0.0981	Torin‐2	A549	1.11	24
8	0.0973	BMS‐536924	MCF7	11.1	24
9	0.0966	Mitoxantrone	A375	0.12	24
10	0.095	DG‐041	A375	40.0	24
11	0.095	AZD8055	MCF7	10.0	24
12	0.095	NVP‐BEZ235	A549	1.11	24
13	0.0943	BRD‐K68548958	MCF7	20.0	24
14	0.0943	Torin‐2	A549	3.33	24
15	0.0935	Torin‐2	A549	0.37	24
16	0.0935	PI‐103	LNCAP	1.11	24
17	0.0927	BRD‐K57080016	A375	80.0	24
18	0.092	BRD‐K98490050	MCF7	10.0	24
19	0.092	GDC‐0980	A549	10	24
20	0.092	PI‐103	A549	10	24
21	0.0912	GSK‐2126458	A375	0.37	24
22	0.0904	Quinacrine hydrochloride	A375	10.0	24
23	0.0897	BMS‐536924	A375	11.1	24
24	0.0897	NVP‐BEZ235	A549	3.33	24
25	0.0889	TG101348	A375	11.1	24
26	0.0889	MK‐2206	HT29	10.0	24
27	0.0889	NVP‐BEZ235	A549	10	24
28	0.0889	GDC‐0941	LNCAP	1.11	24
29	0.0881	BJM‐ctd2‐9	MCF7	10.0	24
30	0.0874	BRD‐K19295594	A375	11.1	24
31	0.0874	NVP‐BEZ235	A549	0.04	24
32	0.0874	GSK‐2126458	A549	0.12	24
33	0.0874	ZSTK‐474	HME1	10	24
34	0.0866	BMS‐754807	A375	10.0	24
35	0.0866	BRD‐K26664453	A375	10.0	24
36	0.0866	TG101348	MCF7	11.1	24
37	0.0866	Dovitinib	A549	10	24
38	0.0858	Wortmannin	A549	10.0	24
39	0.0858	Tyrphostin AG 1478	A375	56.78	24
40	0.0858	WYE‐125132	HME1	3.33	24
41	0.0851	Palbociclib	HME1	10	24
42	0.0843	4‐Demethoxydaunorubicin hydrochloride (65)	MCF7	0.08	24
43	0.0843	Torin‐1	HME1	3.33	24
44	0.0835	Wortmannin	A375	10.0	24
45	0.0835	Torin‐1	HME1	0.37	24
46	0.0828	Wortmannin	PC3	10.0	24
47	0.0828	Nutlin‐3	A375	44.4	24
48	0.0828	PLX‐4032	A375	10.0	24
49	0.0828	DL‐PDMP	HT29	64.0	24
50	0.0828	Mitoxantrone	A375	0.12	24

## 4 Discussion

OC is the second most common gynaecological malignancy and is associated with the highest mortality rate among these cancers due to recurrence, drug resistance and delayed diagnosis [23]. The early diagnosis remains a major challenge as there are no clear signs in the early stages of OC [24]. In the present study, to decipher the dysregulated cellular and molecular mechanisms involved in primary invasive EOC, we used whole RNA sequencing data, as RNA‐seq is the most widely adopted approach for NGKD analysis. RNA‐seq provides comprehensive coverage of gene expression across heterogeneous tissue samples and is useful for identifying cellular and molecular signatures and their associated dysregulated pathways that cause the overall phenotype of a tumour [11]. Here, we used open‐source software, such as ExpressAnalyst and WebGestalt, to perform NGKD analysis of RNA‐seq data on EOC to uncover dysregulated pathways and candidate cancer compounds for primary invasive EOC.

Here, we identified 2123 DEGs in primary invasive EOC compared to benign EOC. GSEA showed that pathways associated with cell division and proliferation were positively enriched. In primary invasive EOC, the cell cycle, DNA replication, control of G1 to S cell cycle and regulation of sister chromatid separation during metaphase–anaphase transition were positively enriched. This could be attributed to the upregulation of different kinases, such as cyclin‐dependent kinases (CDK1 and CDK2), aurora kinases (A and B), polo‐like kinase 1 (PLK1) and DNA damage response kinases (ATM and ATR), as observed in primary invasive EOC. These data highlight the role of primary invasive EOC in rapid cell division and positive enrichment of DNA repair functions; therefore, targeting these newly activated kinase networks in primary invasive EOC should be considered a strategy to inhibit tumour growth.

Similar to other solid cancers such as colorectal cancer (CRC), immune cells in the TME of OC exert a multifaceted influence that can either promote or block tumour progression. The TME of OC is complex and is characterized by macrophages, T cells, natural killer (NK) cells, dendritic cells (DCs) and regulatory T cells (Tregs), which interact with cancer cells and influence disease progression, thereby contributing to antitumor immunity [25]. Since bulk RNA‐seq inherently averages gene expression signals across a heterogeneous mixture of cells, it can mask critical cellular diversity and obscure the presence of rare subpopulations. Tumour‐associated macrophages (TAMs) are abundant in the TME of OC and strongly promote tumour growth by making the TME more immunosuppressive. TAMs interact with other immune cells, such as T cells, and prevent the immune system from attacking tumours. This helps cancer cells to escape the immune system [26] OC cells effectively evade immune surveillance by recruiting immunosuppressive cells, such as Tregs and myeloid‐derived suppressor cells (MDSCs), facilitating cancer progression and metastasis [27]. Hence, to fully understand these intricate gene networks in OC, single‐cell RNA sequencing (scRNA‐seq) should be adopted in future studies.

Moreover, Mitocarta‐based enrichment analysis showed positive enrichment of mitochondrial ribosome (MRC) and OXPHOS subunit (OXPHOS) genes, mtDNA maintenance and mitochondrial central dogma genes. Thus, primary invasive EOC depends on mitochondrial metabolism for rapid development and survival [28]. Immune‐related signalling in our study, such as programmed death receptor‐1 (PD‐1) signalling, NF‐kB signalling, cytokine and chemokine signalling and natural killer cell‐mediated cytotoxicity, was positively enriched. These immune‐activating and immunosuppressive cells are recruited to the TME through interactions between chemokines, cytokines and their receptors. The interaction between DCs expressing PD‐1 and tumour‐infiltrating T cells facilitates immune evasion and contributes to immunosuppression [29]. The changing interactions between immune cells in the TME of OC are important for determining how the disease progresses and developing effective treatment plans. The dual function of immune cells as tumour promoters and suppressors, along with their complex immunoregulatory networks, necessitates the modulation of the TME to enhance antitumor responses [30].

In primary invasive EOC, pathways including ribosome signalling, eukaryotic translation initiation, translation elongation, translation termination and 3′‐UTR‐mediated translational regulation were negatively enriched. Both the general translation machinery and TGF‐beta and Ras signalling pathways were negatively enriched, suggesting that OC cells may selectively downregulate certain ribosomal proteins (RPs) and translation factors to evade stress‐induced toxicity, limit toxic protein aggregation and drive resistance to chemotherapy [31].

RNA‐seq is especially useful for identifying drugs for repurposing based on the gene signatures of a disease that therapeutic agents target to generate a broad, clinically relevant response. Thus, the utilization of existing high‐quality whole transcriptomic datasets provides a powerful foundation for identifying disease‐specific gene signatures and dysregulated pathways suitable for deciphering candidate therapeutic compounds [12]. Based on the NGKD analysis of DEGs in primary invasive EOC, we identified several compounds with antitumor properties that could be effective against OC.

Palbociclib is a CDK inhibitor that can be useful in controlling primary invasive OC [32]. Etoposide is a topoisomerase II inhibitor that causes DNA strand breaks and inhibits DNA replication [33]. Amsacrine (m‐AMSA), a probable DNA intercalator, has been evaluated in a Phase II clinical trial for the treatment of advanced OC, with marginal effects [34]. Wortmannin and AZD‐8055 bind to the PI3K/mTOR pathways and interfere with the signalling of metabolic and survival events within EOC [35]. In addition, compounds such as Naproxen, PP‐110 and BRD‐K65955264 demonstrated to reverse OC‐associated gene signatures based on L1000 FWD and L1000 CDS2 analyses.

## 5 Conclusion

In conclusion, the combination of RNA‐seq and cutting‐edge NGKD methodologies holds significant promise for developing novel therapeutics for primary OC. Several open‐source and commercial software programmes have been developed to process vast amounts of data and help us understand how cancer cells interact with each other and how tumours grow and change over time. These advances in technology and software have enabled the rapid identification of gene signatures in primary invasive EOC. The results of this study could aid in the development of personalized treatment plans based on the unique profile of each tumour type, thus facilitating the development of personalized treatment plans and improving diagnostic and prognostic capabilities in clinics.

## 6 Limitations and Future Directions

The findings of this study depend on NGKD techniques with previously de‐identified datasets from the GEO. As the present study was restricted to bioinformatic methods (i.e., ExpressAnalyst and WebGestalt), we did not perform any in vivo animal modelling or direct human testing to experimentally demonstrate their activity as anti‐cancer agents. The candidate therapeutics identified (palbociclib, etoposide, wortmannin, AZD‐8055 and amsacrine) require further experimental and clinical trials to prove their ability to reverse EOC‐associated gene signatures in these patients. In the future, these bulk RNA‐seq data will be complemented by scRNA‐seq analyses to further elucidate tumour heterogeneity and cellular interactions in the TME. The innovative NGKD approaches presented in this study could be extended to other cancer types, presenting exciting future opportunities for individualized cancer therapies and precision medicine.

## Author Contributions

**Peter Natesan Pushparaj:** conceptualization, writing – original draft, and project administration. **Kalamegam Gauthaman:** writing – review and editing, writing – original draft. **Alaa G. Alahmadi:** methodology, validation. **Reem Nabil Hassan:** visualization, formal analysis. **Hind A. Alkhatabi:** formal analysis, supervision. **Ammar AL‐Farga:** project administration.

## Funding

This research was funded by the Deputyship for Research & Innovation, Ministry of Education in Saudi Arabia, grant number 1045.

## Conflicts of Interest

The authors declare no conflicts of interest.

## Supporting information


**File S1:** This file contains in separate sheets the entire list of 2123 differentially expressed genes (DEGs), ExpressAnalyst outlier report, Differentially enriched pathways using ExpressAnalyst based on KEGG, Reactome, GO BP, GO MF, GO CC, Panther BP, Panther MF, Panther CC, Top 50 L1000 FWD, Top 50 L1000 CDS2, KEGG WebGestalt, Reactome WebGestalt, Panther WebGestalt, GO BP WebGestalt, GO MF WebGestalt, GO CC WebGestalt, WikiPathways WebGestalt, WikiPathways_cancer WebGestalt, Kinases WebGestalt and Mitocarta WebGestalt.

**Figure S1:** ExpressAnalyst Analysis of DEGs based on gene ontology. (A) GO BP (bipartite network), (B) GO MF (bipartite network), (C) GO CC (bipartite network), (D) GO BP (ridgeline plot), (E) GO MF (ridgeline plot) and (F) GO CC (ridgeline plot).
**Figure S2:** ExpressAnalyst analysis of DEGs based on PANTHER. (A) Panther BP (bipartite network), (B) Panther MF (bipartite network), (C) Panther CC (bipartite network), (D) Panther BP (ridgeline plot), (E) Panther MF (ridgeline plot) and (F) Panther CC (ridgeline plot).
**Figure S3:** WebGestalt analysis of DEGs based on (A) KEGG, (B) Reactome, (C) Panther, (D) GO BP, (E) GO MF and (F) GO CC.

## Data Availability

The RNA‐seq dataset used in this study (accession number GSE295399) is openly available in the Gene Expression Omnibus (GEO) for download and reuse.
